# Effects of Vacation Rental Websites on the Concentration of Tourists—Potential Environmental Impacts. An Application to the Balearic Islands in Spain

**DOI:** 10.3390/ijerph15020347

**Published:** 2018-02-15

**Authors:** José María Martín Martín, José Antonio Rodriguez Martín, Karla Aída Zermeño Mejía, José Antonio Salinas Fernández

**Affiliations:** 1 Department of Business, Faculty of Business and Communication, International University of La Rioja, Avenida de la Paz, 137, 26004 Logroño, Spain; 2 Department of Applied Economics, Faculty of Economics and Business, University of Granada, Campus de Cartuja, 18071 Granada, Spain; josearm@ugr.es; 3 Department of Fundamentals of Knowledge, University of Guadalajara, Centro Universitario del Norte, Carretera Federal 23, Santiago Tlaltelolco, Colotlán 46200, Mexico; karla.zermeno@cunorte.udg.mx; 4 Department of Spanish and International Economics, Faculty of Economics and Business, University of Granada, Campus de Cartuja, 18071 Granada, Spain; jasalinas@ugr.es

**Keywords:** Spain, environmental impacts, collaborative economy, peer-to-peer vacation rental websites, seasonality of tourism

## Abstract

The concentration of tourists at certain times of the year can damage sensitive environments. The use of peer-to-peer vacation rental websites has increased greatly during the last decade. This system could either reduce seasonality in touristic destinations where the tourist activity takes place throughout the year at a lower price or on the contrary, it could increase the number of visitors at certain times of the year even more. This paper intends to analyze the effect that these platforms have on tourism seasonality in order to calculate if they help reduce or increase the pressure on the destinations. To do so, the Gini Index has been applied to one of the main touristic spots in Europe, the Balearic Islands in Spain. The conclusion is that this type of accommodation has aggravated the problem, generating a greater concentration of tourists and a higher pressure on the resources of the islands.

## 1. Introduction

Tourism is one of the main driving forces behind economic growth in several countries and world regions [1,2]. Whereas the world-leading economic powers obtain huge benefits from tourism, developing countries rely on tourism to consolidate their economic growth. As presented in the United Nations Environment Program [3], tourism, as with any other economic activity, generates both positive and negative impacts on the environment, society and economy at local, national, and global scales. There are also pros and cons relative to the environmental effects since tourism contributes to preserving environments in numerous regions. However, international organizations and academic papers have both suggested the negative impacts generated by tourism [4,5,6,7,8].

Tourism seasonality does not exist exclusively in the tourism sector, even though it is the sector where it has a stronger presence. Focusing on this sector and acknowledging that there is not a widely accepted definition, we take Butler’s definition as a reference [9] “a temporal imbalance in the phenomenon of tourism, which may be expressed in terms of dimensions of such elements as numbers of visitors, expenditure of visitors, traffic on highways and other forms of transportation, employment, and admissions to attractions”. Several studies have analyzed the cause of tourism seasonality, for instance, Hylleberg suggested the following set of factors: weather (e.g., temperature, hours of sunshine), calendar effects (e.g., timing of religious festivals such as Christmas, Easter, Eid or Vesak) and timing decisions (e.g., school vacations, industry vacations, tax years, accounting periods, dates for dividend and bonus payments, etc.) [10]. As discussed below, the uneven pattern of the economic activity throughout the year has many effects on tourist destinations. The environmental impacts derived from a high concentration of visitors in short periods of time are among these effects. 

The collaborative economy concept, as far as tourism sector is concerned, usually refers to several activities, such as house swapping, house renting, ridesharing, voluntourism, couchsurfing, dinner hosting and similar innovations that epitomize the collaborative economy [11]. This phenomenon, also known as collaborative consumption, the sharing economy and peer-to-peer consumption, emerges as a consequence of changes recently experimented by society, economy and technology. Such changes include the possibility of accessing temporarily to certain assets, the development of transactions between producers and customers mediated online, the setting of relationships between host and local population, and new forms of interaction between consumers that allow them to share information in order to limit the possible risks [11]. In the tourism sphere, collaborative economy companies adopt both for-profit and not-for-profit structures. This has disrupted the industrial systems related to tourism around the world [12]. Supporters and opponents of the collaborative economy within tourism scope are both very numerous. The supporters defend the value that collaborative economies has since it makes it possible to exploit social and economic resources not exploited before from a sustainable and decentralized point of view [13]. On the contrary, opponents warn about the illusion of participation, crowd power and customer sovereignty [14]. This new type of tourism planning poses a challenge for both planners and policymakers in areas with a big rise in the number of tourist arrivals linked with this trend. Problems such as neighborhood nuisances, traffic, and scarcity of parking spots may be associated with this trend [15]. 

The growth of peer-to-peer vacation rental platforms such as Airbnb, Homelidays, and Wimdu has altered the tourism context. The studies into collaborative economy tourism accommodation platforms, which are more and more frequent in the past few years, cover issues such as marketing, profitability, public image of the destination, etc. However, there is very little bibliography on the impacts derived from tourism. Some studies highlight the impacts that are predictable, as Dredge et al. points out: “Local residents may be directly or indirectly impacted by the collaborative economy tourism accommodation sector.” As Dredge et al. [11] point out, “there are residents in neighboring houses and apartments that must deal with local impacts (e.g., noise and nuisance caused by tourist behavior, loss of community cohesion, impacts of community facilities, impacts on rental and property prices, etc.)”. In the same study, focused on Barcelona, Paris and Berlin, it is argued that the increase of the amount of accommodation for tourists may have directly caused a rise in property prices of centrally located districts. Moreover, the rise of the collaborative economy has also coincided with housing shortages and affordability issues. Other disturbances have also been detected, such as the intensification of tourist concentration in certain spots, noise and nuisance caused by tourist behavior, loss of community cohesion, impacts of community facilities and impacts on rental and property prices, etc. Sellares et al. [16] highlights the effect this type of tourism accommodation has on the local people with regard to the increase of rental prices and a loss of quality of life. The effects that this new tourism structure has on the seasonal concentration of tourists and the consequences in destinations where seasonality is already severe have not been paid any attention yet. 

This paper provides an analysis that has not been carried out up until now. It offers data on the number of tourists that take part in a collaborative economy tourism accommodation platform in environmentally sensitive destinations. Therefore, this research aims to determine whether the annual number of visitors staying in this type of accommodation improves seasonality or aggravates it. The former is backed by an increase in the amount of accommodation offered outside of the peak season and its lower price, enabling tourists to travel more times throughout the year apart from their main trip. Nevertheless, the demand linked to this type of accommodation offer could exacerbate the patterns of concentration of visitors as a consequence of the unplanned increase in the number of bed places, thus aggravating a problem that already exists. Choosing the Balearic Islands as the case study is justified for many reasons. It is one of the main touristic spots in Europe and the Spanish region with the highest seasonality level [17]. Moreover, the tourists that arrive at the islands are very sensitive when it comes to the prices and travel expenses [18], thus the effect that a more affordable accommodation offer has on the prolongation or shortening of the tourist season must be determined. 

This paper begins with an exposition of the environmental risks derived from tourism seasonality and the rise in the number of tourists. Secondly, we analyze meticulously the area relative to the case study, and shows the ecological consequences that are generated by the current tourism model of the Balearic Islands. The first section is developed through an exhaustive review of the literature on tourism seasonality, tourism impacts and sustainability. In the second part, we also work keeping in mind the bibliography on tourism sustainability, especially focused on the papers applied to the Balearic Islands. In the following section, we show and justify the methodology we have chosen to complete the analysis proposed below, as well as the sources of information that give meaning to this study. Later, the main results are presented. The results show in a direct way the effect that tourism demand associated with touristic rental properties rented online has on seasonality. The last two sections, which concern conclusions and discussion, analyze the risks derived from these trends according to the results obtained. Finally, we propose future possible lines of research.

## 2. Risks Derived from Tourism Seasonality and Increase in the Number of Arrivals

In Spain, a country with a clear tourism profile, the rise of the seasonality level has gone hand in hand with the growth of mass tourism, linked to the so-called “sun and beach” tourism. This has caused the destinations with a larger number of visitors to become more seasonal [17], and shows a clear risk for the natural environment where tourism is developed. There are several impacts derived from seasonality. These impacts will be different depending upon the configuration of the destination due to the fact that the causes and intensity of seasonality are also linked to certain characteristics of the destination [19]. The negative tourism effects are sorted into four different categories: ecological, economic, sociocultural and labor [20]. Seasonality generates negative effects related to economic and labor impacts during the valley seasons, where arrivals are drastically reduced. While the environmental and sociocultural impacts are related to the excessive number of visitors at certain times of the year, environmental impacts concern the massive number of visitors during the peak season. Among these effects are included the congestion of rural lanes, disturbance of wildlife, physical erosion of footpaths and litter problems [21]. Tourism can generate a high pressure on the carrying capacity of some destinations due to the exhausting use of its resources during peak seasons [22]. Butler [9] concludes that one of the main problems of seasonality is the impact derived from the pressure put on the environment due to the overcrowding and overexploitation of the natural spaces. The ideal scenery would have a homogeneously distributed activity level throughout the year so that the pressure on the resources is reduced. In destinations with a higher concentration of visitors, a resting period understood as a time for the recovery of the natural resources and a return to normality is justified [22,23,24]. This implies, therefore, that the seasonal economic and labor impacts would be increased since they are bound to a period with little activity or none at all. On top of that, little would be done to restrain the negative effect that a peak season has on the natural resources and the environment.

Tourism also produces another set of interferences on the environments where it is developed. These interferences are derived from the number of arrivals, a factor that can also intensify the effects linked to seasonality. Plenty of developed countries have followed for years the same tourism model, which consists of attracting the largest number of visitors possible during the longest period possible [25]. It is because of this that sustainable tourism models have shifted from understanding tourism not only as an economic effect to understanding it as an environmental and social effect as well [26].

This paper intends to analyze seasonality within a context of growth where the demand linked to the increase in peer-to-peer touristic rentals takes place in a collaborative marketplace. The aforementioned factors—the increase in the number of visitors and the development and use of collaborative economy platforms—are co-related. This is because the development of the collaborative economy takes place in a context in which European cities experience the impacts of years and years of pro-growth strategies, encouraged by the consolidation of low-cost mass tourism [15]. In a moment where low-cost airlines and affordable accommodations boost the tourist demand, we should pay more attention to the actual pressure put on environmental resources, which is intensified at certain times of the year. The high concentration of visitors could surpass the time of recovery of resources and wastes allocation. As pointed out by Cazarro [27], regarding the excessive use of water, “the largest kind of tourism in Spain is that of ‘sun and beach’, concentrated in arid and water-stressed regions, some of which have already needed water transfers to prevent salinization”.

## 3. Ecological Challenges Derived from the Pressure of Tourism in the Balearic Islands.

The Balearic Islands, located on the east shore of Spain, are one of the main tourist destinations in Europe. Their geographical situation provides them with several advantages over others. It is possible to reach the islands in less than four hours from almost any European country. Tourism prevails over the other economic sectors and accounts for the 85% of the Gross Domestic Product (GDP), thus operating profound changes that have transformed an area with a rural economy into one of the richest in Spain [18]. The islands cover an area of 5040 square kilometers and they have 1428 km of coast. This archipelago is formed by three large islands: Mallorca, Menorca, and Ibiza; and two others of smaller size: Formentera and Cabrera (much less exploited than the former). 

The tourism developed on the islands is quite controversial among the local people. Although it accounts for the largest part of the islands’ income, it also causes many negative impacts. Regarding the management of the environmental impacts, the large number of visitors is both a success and a challenge. In 2016, the Balearic Islands hosted a total of 18.3 million tourists, 40% more than in 2008 [28]. The islands have one of the highest tourist-per-capita ratios of the world—16:1—taking the local population into consideration. Besides the problems caused by the growth in the number of arrivals, this destination must also manage the challenge of dealing with the highest seasonality level of Spain [17]. This archipelago has devoted its tourism offer to the so-called “sun and beach tourism”, and unlike other areas with a more stable climate throughout the year, the Balearic Islands depends heavily on the summer. If we take data concerning 2016 into account, it can be observed how 50% of the arrivals (more than 9 million visitors) took place during the months of June, July and August [28]. Apart from the tourism floating population, we must add up the employees of the tourism sector who arrive every summer to the islands from different places of Spain to cover the job vacancies. The number of people working as employees of the tourism sector reaches an average of 176,727 during the summer, while in the off-season, the number falls to 73,198 employees [28]. The increase in the demand and a large number of visitors result in a complex situation when trying to manage potential ecological impacts. 

From an ecological perspective, managing solid waste is one of the main challenges caused by the growth of tourism [29]. Several studies have pointed out the large increase of solid waste generation as a consequence of the seasonal population in tourist areas [30,31,32,33]. Therefore, in these areas, it is especially important to collect, transport, process and finally dispose of solid waste in an environmentally sound and cost-efficient way [31]. Mateu et al. [34] argue that, according to estimations, if the tourist population grows by 1%, the solid waste generation increases by 0.282%. Moreover, this does not just happen during the stay of the tourists on the islands, but for a long time after they have left. Equally, it is estimated that each tourist produces 1.31 kg of solid waste per day, which comes close to the 1.48 kg produced by each local.

Tourism also puts a high pressure on water resources in ecosystems with already limited resources such as the Balearic Islands. Overcrowding may have severe consequences in environmentally sensitive areas, especially coastal zones and islands [35,36]. Tourism tends to have distinct seasonal variations and to be concentrated in regions that are often associated with the limited availability of water resources [37]. The success of tourism experiments in the Balearic Islands results in the overcrowding of beaches during the warm season. This causes a peak in the consumption of water resources that overlaps with the dry season. This situation implies an over-extraction and lowering of the groundwater [38]. The consequence of this over exploitation is the incapability to reach the necessary resupply rate of aquifers. Inland aquifers have lost 55 m of their total in 15 years because of the extraction, which surpasses the annual recovery rate. In an average year, the total available fresh water in Mallorca is 250,106 cubic meters. In 1990, the annual demand for water already exceeded that volume. Desalination plants were built to make up for this deficit, which has increased the volume of water available, but in exchange it has also caused a rise in its price, as well as increasing the energetic cost and pollution levels [39]. 

In the academic literature, there are many additional references concerning the different economic instruments proposed to internalize the costs and the environmental implications of tourism in the Balearic Islands [40,41]. Some studies have analyzed the characteristics of tourism demand, visit trends and seasonal patterns [42,43], but the effect of the peer-to-peer accommodation rental websites has not been studied yet. The results of this study will enhance the global understanding of this new system of intermediation in addition to providing conclusions focused on this region.

## 4. Methodology

The basic aim of this research is to determine whether the tourism demand linked to collaborative economy tourism accommodation websites increases or reduces tourism seasonality. To reach this goal, two elements are required: an appropriate measurement system of the concentration of visitors and monthly disaggregated data from each type of accommodation.

Papers of the academic literature address the issue of seasonality from different perspectives. Some of them are focused on breaking down the seasonal factors that constitute the time series, i.e., [1,44,45,46,47,48]. Other papers prefer to develop predictions or modeling [49,50,51,52,53,54,55,56,57,58]. Quantifying the levels of concentration of visitors through indexes represents one of the main lines of work regarding seasonality [43,59,60,61]. Nevertheless, there is not a widely accepted method to measure seasonality’s intensity [20]. The most widespread procedures to quantify this measurement are focused on the application of indexes, such as the Gini Index (GI), the Theil Index and the Coefficient of Variation since they are able to offer a unique measurement of the concentration in one year. 

The GI is the most commonly used in this type of analysis [59]. This index fulfills the Pigon-Dalton condition, whose interpretation applied to tourism seasonality implies that the transfer of the supply or demand from a month with a higher number of arrivals to another with a lower number diminishes the coefficients, or so to say, seasonality [62]. Wanhill [61] recommends the application of this methodology over other alternatives, since it takes the biases of distribution into consideration and is less influenced by extreme values. Lundtorp [60] shows that the GI is the most stable seasonality measurement system. Moreover, choosing this system to measure the concentration of visitors in this paper is justified because in the last 30 years of academic literature, the GI has been the tool mostly used to reach this end [60,61,63,64,65]. The GI, usually applied to the number of arrivals, measures the degree of imbalance of tourism throughout the year [66]. The GI is built using the Lorenz Curve, which shows the cumulative frequency range of observations, starting off at the lowest number [60]. The Gini coefficient equals the area between the Lorenz Curve and the 45 degrees line that divides the area below the line and is expressed as follows:

$$GI=1+\left(\frac{1}{n}\right)-\left(\frac{2}{\left(n^{2}\cdot `x\right)}\right).\left(x_{1}+2x_{2}+3x_{3}+\ldots nx_{n}\right)$$

In this formula, *n* is the number of months (the study is performed using monthly data), *x*_1_, *x*_2_, *x*_3_, …, *x_n_* represents the individual observations in descending magnitude order and *`x* is the average number of observations [67]. The maximum value of the index is 1 and it implies that the total of visitors of the year concentrates in one month, whereas the minimum value, 0, implies an equal distribution of the arrivals every month. 

Once the measurement method has been defined, the second necessary element to reach the goal is locating monthly disaggregated data. We rely on data provided by the Regional Government of the Balearic Islands via tourist data yearbooks [28]. This institution provides monthly data that allows us to differentiate the many types of accommodations where tourists stay. The Regional Government releases these statistics using several sources of information. One of these methods consists of face-to-face surveys, where they ask tourists in which type of accommodation they decided to stay. Following this procedure, which takes a representative sample into consideration, we extrapolate the information relative to the tourist arrivals to the islands so that we can obtain data for each type of accommodation. Since collaborative economy online accommodation platforms are quite new, these statistics were released in 2016 for the first time. Surveys carried out in a direct way guarantee that the data obtained comprehend the entirety of the collaborative economy tourism accommodation platforms. All data used for this study have their origin in official statistics released by the Regional Government of the Balearic Islands. We have added the data necessary to build the categories with which we work and shown below. 

We work with three different points in time: 2016 because it was the last year with complete and definitive data, 2008 as the year the main website, Airbnb, started its activity; and 2012 as a halfway checkpoint. The regulated tourism accommodation offer (hotels, aparthotels, pensions and camping sites) will be compared with the non-regulated offer (tourists accommodated in private properties, friends or family’s properties and rental properties). In 2016, aside from the non-regulated offer, it is also possible to count with disaggregated data regarding the properties rented for touristic purposes using online platforms. Using these data, we make a comparison between the three different categories, firstly in absolute values and afterwards according to their respective GI. We propose the respective analyses of the GI referring to the number of tourists staying in regulated accommodation and its evolution, the analysis of the GI concerning the number of tourists staying in non-regulated accommodation and its evolution; the analysis of the GI regarding the number of tourists staying in touristic accommodation rented using online rental platforms and their comparison with the global GI. Thanks to these analyses, we can draw conclusions as to the effect this new type of organization of the tourism offer has on the concentration of tourist demand and the deseasonalization of destinations. Both factors determine the pressure that tourism has on the environment. 

## 5. Results

The first thing that stands out in how tourism has evolved in the Balearic Islands is the huge increase in the number of tourists who visit the destination every year (Table 1). In 2016, the arrivals exceeded 18.3 million, 40% more than in 2008. This figure alone represents a challenge due to the ecological impact derived from the extra population that the islands must host. According to the type of accommodation chosen by each tourist, we can draw additional conclusions from the evolution of the arrivals. In 2008, the tourists staying in regulated establishments (hotels, aparthotels, pensions and camping sites) made up 73% of the total, a percentage that had decreased to 66.9% in 2016 (Table 2). In absolute terms, the number of people that stay in this type of establishment has risen to a total of 28.43%. The number of tourists accommodated in non-regulated accommodation (private properties, friends/family’s properties and rental properties intermediated online or offline), went from a 27.0% of the total in 2008 to a 33.1% in 2016. Moreover, the number of tourists using this type of accommodation has grown, in absolute terms, by 71.8% percent in the last few years, which has consolidated a trend with important repercussions for the tourism context of the Balearic Islands and the local residents. Since there are not disaggregated data for 2008 and 2012 as per type of intermediation, these data include both tourists using websites to rent properties and those using traditional methods.

Once the number of tourists staying in touristic rental properties and its evolution are determined, their effect on the Balearic Islands’ seasonality is analyzed. The data expressed in absolute values in Table 3 show the concentration of visitors during the peak months: June, July and August. In 2016, 47.5% of the tourists accommodated in regulated establishments visited the islands in those months (hotels, aparthotels, pensions and camping sites). The number goes up to 52.9% if we take the non-regulated establishments into consideration (private properties, friends/family’s properties and rental properties intermediated online or offline). The percentage of tourists accommodated in properties rented via collaborative economy tourism accommodation platforms during the months of June, July and August is 56.6%. Therefore, these waves of tourists contribute to increasing seasonality, especially within the type of tourism that has experimented a bigger increase in absolute values, which is why the problem of overcrowding is becoming more intense.

The analysis provided by the GI describes how the growing number of tourists staying in properties rented through websites affects seasonality (Table 4), according to the pattern of their arrivals throughout the year. Given that the monthly data concerning the number of tourists staying in rental properties using online websites belong only to 2016, it is not possible to analyze the evolution of this kind of visitor. Looking at last year, the degree of concentration of tourists is higher in properties rented online than in any other type of accommodation. For that matter, if we compare 2008 and 2016, we can observe how the GI calculated for the number of tourists staying in regulated establishments has decreased from a value of 0.48 to 0.46. In the case of non-regulated establishments, after a slight increase of the concentration in 2012, the GI went down from a value of 0.475 to 0.444 in 2016. The latter figure shows a lower level of seasonality compared with the level that refers to the tourists who were accommodated in regulated establishments. As far as the data of regulated establishments are concerned, seasonality seems to be restrained in the Balearic Islands. The evolution of the annual distribution of tourists staying in non-regulated accommodation as a whole contributes to reducing seasonality as well. Therefore, the factor increasing seasonality in the Balearic Islands is the annual distribution of the total number of tourists staying in touristic properties rented using a website. The growing weight of this type of accommodation has contributed, generally, to counteracting the reduction of the levels of seasonality attributed to the other forms of accommodation. The GI of the touristic rental properties (rented via online) comes to 0.55, whereas the GI related to the rest of accommodations is 0.453, which would rise to 0.482 considering the total number of tourists. Thus, the demand associated with accommodation rented through a website contributes to increasing seasonality in the Balearic Islands, belittling achievements previously accomplished. Moreover, the fact that it is the modality that has grown the most, the expected outcome is even worse. The GI related to the seasonality of the offer of regulated accommodation in Spain is given as a reference. In 2016 it was 0.18, which shows the important problem that is taking place in the Balearic Islands.

## 6. Discussion

The results obtained point out the urgent need to incorporate the supply variable relative to non-regulated touristic properties to processes of destination planning. It has been confirmed that the flow of tourists staying in holiday properties mediated online contribute to increasing seasonality, which is the basis of this study. Combining an increase in the level of seasonality with a large increase in the number of arrivals will undeniably intensify the pressure put on natural resources. This pressure in environmentally sensitive destinations, with water scarcity or a limited waste management results in a huge challenge for public planning. This is particularly the case of the Balearic Islands, a region that suffers from high tourism seasonality, which is now being worsened as consequence of these new types of tourism intermediation. Previous studies show the positive benefits that tourism has on this region. We broaden the conclusions of said studies with our current analysis since it warns about the danger that these tendencies constitute for the environment. 

The analysis of data and its recent evolution shows us how the concentration of tourists accommodated in regulated establishments began to reduce. This is possibly due to the development of policies of diversification within the hotel industry. These improvements are being overridden because of the effect of tourist flows associated with the offer of accommodations rented online. These properties are left out of professional proceedings that manage the offer of accommodations. Thus, they do not reach the same success in the deseasonalization of the destination. Instead, the effect is completely the opposite, just as shown in this study. 

## 7. Conclusions

It can be objectively asserted that the modality comprised of touristic rental accommodation is now more present than before compared to other types of travel planning. The expansion of collaborative economy tourism accommodation platforms has also contributed to it. This change raises an issue in the legislative planning of several locations, which have not been modernized yet, thus excluding the real number of accommodation places from a coherent process of planning according to the carrying capacity of the destination. The growth in tourism has been facilitated by public and private policies that prioritized the increase of arrivals over other parameters, which does nothing but worsen the planning problem. The third element conditioning the impact of tourism is the concentration of visitors at certain times of the year because it increases the pressure on natural resources [68]. A high level of seasonality conditions the support of the local residents towards tourism given that the local community shapes their attitude towards tourism after having evaluated its potential benefits and negative impacts, which are intensified due to seasonality [69]. As argued by Peric et al. [70], there is a gap in the knowledge and understanding of mechanisms on how to deliver social and economic community benefits. Sustainable tourism aims to channel tourism to the advantage of all stakeholder-destination places and communities, tourists and all the associated activities and services [71].

Environmentally sensitive spots such as the Balearic Islands face the challenge of managing growing flows of tourists concentrated in a short period of time. The challenge is magnified if we take into consideration that the increasing accommodation offer eludes public planning in terms of quality conditions and number of vacancies. The pressure put on the resources is intensified due to the impact of the increasing tourist arrivals and their concentration. When it comes to the Balearic Islands, it affects directly the water consumption and the aquifers’ capacity to recover, the increase of environmental pollution, waste production, etc. All of this pushes to its limits a sensitive environment that relies heavily on nature to uphold tourism.

The annual data on the flows of visitors conclude that the tourist arrivals associated with regulated establishments and owned properties are losing ground with respect to touristic establishments rented online. The demand linked to this type of accommodation becomes more concentrated during the peak season compared with other types of accommodation, which increases tourism seasonality and the pressure put on the destinations. In fact, the increase in the number of users of this system overrides the improvements made by other modalities in terms of seasonality. This being so, the public sector should limit the places offered in accommodation up for rent during the peak season to reduce pressure on the destinations while promoting them during the valley season. The plunges in activity during the valley season generate equally severe problems since they force the local population to seek alternative sources of income to those associated with tourism [72]. It is clear that the lack of planning can result in an increase in the number of visitors staying in this kind of accommodation as well as in an increase of the concentration of tourists and a higher pressure on the environment.

The limitations of this research are related to the novelty of the object of study given that intermediating touristic properties via online platforms is a recent phenomenon. Therefore, official statistics are undergoing a process of adaptation in order to include data referred to this housing system. This limits the possibility to perform evolutionary studies. The paper is also limited by the low number of regions that offer this kind of statistics, which reduce the number of comparisons possible. Other types of accommodation, such as house exchange, are left out of these statistics because they do not consider this modality on its own. Finally, we must point out how useful it would be to count with local statistical data that allow for the understanding of the problem depending on the type of geographical area (coastal, urban or rural).

This study brings to light the necessity of the public sector to take part in planning the offer of properties intermediated via online platforms. Thus, guaranteeing a number of places suitable for the carrying capacity of the different destinations. This planning should be developed hand in hand with the hotel industry. The rapid growth of this type of accommodation has resulted in the necessity of updating the legislation as well as including these accommodations in the processes of tourism planning in order to restrain their growth. This study suggests that the planning must have two different approaches, from the point of view of space (to avoid geographic concentration clusters) and time (adjusting supply throughout the year). Many segments of the local population might also benefit economically from this type of accommodation, which puts to use social and economic resources that are underused. Moreover, it can generate new forms of social interaction that enrich both the tourists and the local population. Therefore, to achieve this goal of preserving potential benefits while assuming the necessity of limiting negative impacts, a bigger effort is required in public planning. Due to the novelty of these systems of intermediation, this effort has not been made yet. 

This line of research should be completed with studies analyzing the impact of several legislative regulations on the planning of touristic rentals. It is also necessary to know the way in which the concentration of visitors affects the quality of life of local communities, since an increase in the number of tourist arrivals at traditionally residential zones can generate many interferences, even more in destinations that suffer from a high seasonality. To restrain these interferences, a mean of participation should be established so that the local residents are able to participate in public planning processes. This would lead the way for new and interesting lines of research. 

## Figures and Tables

**Table 1:** Evolution of the number of tourist arrivals to the Balearic Islands as per type of accommodation. Years 2008, 2012 and 2016.

Year	Total Number of Tourists	Tourists Accommodated in Regulated Establishments	Tourists Accommodated in Non-Regulated Establishments
2008	13,103,901	9,565,848	3,538,054
2012	15,346,663	10,589,197	4,757,465
2016	18,363,889	12,285,442	6,078,447

Source: Agency for Tourism of the Balearic Islands. Own elaboration.

**Table 2:** Percentage distribution of tourist arrivals to the Balearic Islands as per type of accommodation. Years 2008, 2012 and 2016.

Year	Tourists Accommodated in Regulated Establishments	Tourists Accommodated in Non-Regulated Establishments
2008	73.0%	27.0%
2012	69.0%	31.0%
2016	66.9%	33.1%

Source: Agency for Tourism of the Balearic Islands. Own elaboration.

**Table 3:** Monthly distribution of tourist arrivals to the Balearic Islands sorted by accommodation chosen. Years 2008, 2012 and 2016.

Year	January	February	March	April	May	June	July	August	September	October	November	December
Tourists accommodated in regulated establishments												
2016	246,917	130,729	516,686	1,039,526	1,927,474	1,713,061	2,014,975	2,117,470	1,538,135	821,721	139,193	79,555
2012	61,117	158,757	339,243	536,913	1,143,608	1,691,798	2,062,804	2,009,826	1,637,077	792,183	102,033	53,839
2008	95,099	163,881	314,237	638,952	1,175,997	1,434,831	1,814,803	1,676,477	1,272,543	738,664	160,552	79,811
Tourists accommodated in non-regulated establishments												
2016	181,054	124,155	155,081	390,133	619,641	1,046,782	1,046,782	1,126,503	652,502	483,625	132,030	120,157
2012	156,069	96,528	162,094	269,709	291,289	530,093	908,425	1,089,635	684,131	372,877	105,573	91,041
2008	132,931	94,415	132,188	274,385	327,950	342,409	528,198	738,933	481,178	293,412	103,551	88,503
Tourists accommodated in regulated and non-regulated establishments												
2016	427,971	254,885	671,767	1,429,660	2,547,115	2,759,842	3,061,756	3,243,973	2,190,637	1,305,347	271,223	199,712
Tourists accommodated in online rented properties												
2016	22,177	20,086	36,230	135,837	229,520	338,325	523,666	526,670	378,942	211,215	21,298	16,796

Source: Agency for Tourism of the Balearic Islands. Own elaboration.

**Table 4:** Gini Index of the monthly tourist arrivals to the Balearic Islands. Calculated by type of accommodation. Years 2008, 2012 and 2016.

Type of Accommodation	2016	2012	2008
Tourists accommodated in regulated establishments	0.463	0.522	0.480
Tourists accommodated in non-regulated establishments	0.444	0.475	0.395
Tourists accommodated in regulated and non-regulated establishments	0.453		
Tourists accommodated in online rented properties	0.550		

Source: Agency for Tourism of the Balearic Islands. Own elaboration.
